# AutoSOUL: Deep Convolutional Neural Network for Autonomous Selection of Ultrasound Speckle Tracking Parameters

**DOI:** 10.1109/TUSON.2026.3651730

**Published:** 2026-02

**Authors:** Md Ashikuzzaman, MD Jahin Alam, Ahmed El-Desoky, Eniola Oluyemi, Kelly Myers, Emily Ambinder, Muyinatu A. Lediju Bell

**Affiliations:** Department of Electrical and Computer Engineering, Johns Hopkins University, Baltimore, MD 21218 USA; Department of Electrical and Computer Engineering, Johns Hopkins University, Baltimore, MD 21218 USA; Department of Electrical and Computer Engineering, Johns Hopkins University, Baltimore, MD 21218 USA; Department of Radiology and Radiological Science, Johns Hopkins Medicine, Baltimore, MD 21287 USA; Department of Radiology and Radiological Science, Johns Hopkins Medicine, Baltimore, MD 21287 USA; Department of Radiology and Radiological Science, Johns Hopkins Medicine, Baltimore, MD 21287 USA; Department of Electrical and Computer Engineering, the Department of Biomedical Engineering, the Department of Computer Science, and the Department of Oncology, Johns Hopkins University, Baltimore, MD 21218 USA

**Keywords:** Automatic parameter selection, convolutional neural network (CNN), deep learning, speckle tracking, ultrasound strain elastography

## Abstract

In medical diagnostics, ultrasound strain elastography offers a noninvasive method to assess tissue stiffness, aiding in distinguishing tumors, cysts, and benign lesions from healthy tissue. A strain elastography framework employs a speckle-tracking technique to estimate the displacement between precompression and postcompression radio frequency (RF) frames, which is spatially differentiated to calculate the strain field. Second-order ultrasound elastography (SOUL) is a recently published speckle-tracking algorithm that optimizes a regularized cost function comprising data fidelity and regularization terms. However, the success of SOUL to produce high-quality strain images depends on the manual selection of regularization parameters, making it user-dependent and limiting its efficiency and usability. To mitigate this manual parameter selection, herein, we propose AutoSOUL, a deep convolutional neural network (CNN)-based framework, to autonomously select the optimal regularization parameters of SOUL. A custom-designed CNN was trained on simulated datasets and fine-tuned on phantom datasets to classify a strain image as acceptable or unacceptable and predict the optimal strain image and parameter set corresponding to a given precompression and postcompression RF frame set. When tested on simulated, phantom, and in vivo breast datasets, the ranges of the accuracy, precision, recall, and F1-scores produced by the trained CNN model were 77%−99%, 68%−98%, 97%−100%, and 81%−99%, respectively. The RMSE, MAE, SNR, CNR, and SR values calculated from the optimal strain images suggested by AutoSOUL were within 1.5%−4%, 6%−9%, 0%−30%, 0%−21%, and 0%−6%, respectively, of values obtained from the corresponding manually tuned strain images. AutoSOUL reduced the execution time of the otherwise required manual tuning and selection process of SOUL by a factor of 84.4. These results indicate that AutoSOUL has the potential to enhance the usability of ultrasound elastography, facilitating more efficient clinical evaluation of tissue abnormalities.

## Graphical Abstract

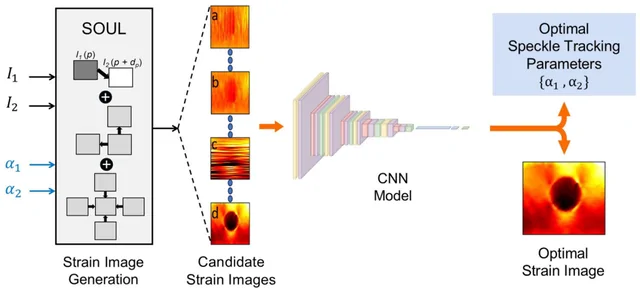

## I. Introduction

Ultrasound elastography [1], [2] is a noninvasive medical imaging technique that evaluates tissue stiffness to aid in diagnosing various pathologies (e.g., tumors, cancers, benign lesions, and cysts), with the understanding that certain pathologies alter tissue stiffness [3]. Among different ultrasound elastography techniques, such as strain elastography [4], [5], [6] and shear wave elastography [7], [8], [9], [10], [11], [12], strain elastography applies pressure to a tissue of interest using a handheld probe that acquires time-series ultrasound frames during tissue deformation. A speckle-tracking technique estimates the displacement field between a pre- and postdeformed radio frequency (RF) frames. The estimated displacement field is spatially differentiated to render the strain map. Applications of ultrasound strain elastography include breast health assessment [13], [14], [15], [16], liver tissue characterization and fibrosis staging [17], [18], [19], [20], [21], cardiovascular [22], [23] and neural [24], [25] health monitoring, myofascial dysfunction quantification [26], [27], [28], [29], [30], and kidney [31] or thyroid [32] pathology assessments.

Tracking the displacement field between pre and postdeformed ultrasound frames is a critical step in ultrasound elastography, as it directly impacts the accuracy of the estimated elastogram (i.e., strain image) [33]. However, ultrasonic displacement tracking is an ill-posed problem due to the nature of ultrasound data (e.g., numerous samples exhibiting identical amplitudes, shared characteristics, and similar patterns) [13], [34], [35]. To overcome this challenge, window-based displacement tracking algorithms [36], [37], [38], [39] divide ultrasound frames into multiple data windows and impose the constraint that all samples within a window share the same displacement. Based on this assumption, these methods estimate displacement by identifying the best-matching postdeformation window using normalized cross-correlation [40], [41], [42], [43], [44] or zero phase crossing [45], [46], [47], [48]. While relatively straightforward to implement, window-based algorithms are known to be noise-sensitive [49] and involve a trade-off between accuracy and resolution depending on window size [50], [51]. Deep learning-based techniques [49], [52], [53], [54], [55] were recently introduced to learn statistical patterns and correlations in pre and postdeformed ultrasound data for displacement tracking. During testing and deployment, these methods primarily rely on inference and GPU acceleration, making them well-suited for real-time applications. However, large amounts of training data are required to develop accurate models [53], and the limited availability of clinical datasets remains a key challenge to broader adoption in ultrasound displacement tracking.

Alongside window-based and deep learning-based techniques, energy function optimization [56], [57], [58], [59], [60], [61], [62] is a widely used approach for ultrasonic displacement tracking. This approach models tissue deformation as a nonlinear cost function and estimates displacement by optimizing this cost function, which typically includes data amplitude fidelity and continuity terms [17], [63], with the latter often incorporating displacement regularity and tissue deformation mechanics [5]. By simultaneously considering ultrasound data and smoothness constraints, energy function optimization balances estimation accuracy and smoothness within a unified framework [19]. However, a major drawback of this approach is its high computational complexity. Dynamic programming [64] addressed this computational challenge by introducing an adaptive search range and utilizing stored intermediate computations to improve efficiency. However, dynamic programming-based displacement estimation is limited to integer values. To achieve subpixel resolution, dynamic programming analytic minimization [17] refined the dynamic programming integer estimates per A-line by analytically optimizing a cost function comprising data and continuity terms. Despite this improvement, strain estimates suffered from vertical streak artifacts due to independent displacement estimation along each A-line.

To eliminate streak artifacts, global ultrasound elastography (GLUE) [65] was introduced, simultaneously estimating the displacements of multiple samples within an RF frame. However, GLUE formulates its continuity term using only first-order displacement derivatives, which does not fully capture the mechanics of tissue deformation in ultrasound strain elastography. As a result, GLUE-generated strain images tend to be noisy and low in contrast. To overcome this limitation, second-order ultrasound elastography (SOUL) [66] was developed, incorporating both first- and second-order displacement derivatives to better-model tissue mechanics. SOUL demonstrated strong potential to generate high-quality axial strain images [5], [19], [66]. However, its success heavily depends on properly selected regularization parameters. Currently, SOUL requires manual tuning of its regularization weights to produce high-quality strain images, making it highly user-dependent, which limits efficiency and usability.

In the context of breast ultrasound elastography, manual adjustment of the parameters required by SOUL may pose significant challenges. Breast tissue exhibits complex and heterogeneous properties [67], [68], [69], varying across patients and lesion types. The need for manual adjustment of regularization weights makes the technique highly operator-dependent, introducing variability in strain image quality and limiting reproducibility. This inconsistency can hinder the reliable differentiation of benign and malignant lesions, reducing the clinical utility of SOUL in diagnostic settings. Automating the parameter selection process is critical to improving robustness, methodological consistency, and reproducibility of breast ultrasound elastography.

Herein, we propose training a custom-designed convolutional neural network (CNN) to automate the parameter selection process in SOUL, naming the process AutoSOUL. Several factors motivate the use of a CNN for this task. First, evaluating the acceptability of a strain image is inherently a classification problem, and CNNs have consistently demonstrated state-of-the-art classification performance across various domains [70]. Second, CNNs require no additional hardware and can be seamlessly integrated into clinical ultrasound systems. Third, CNNs are computationally efficient during inference, enabling real-time implementation. While CNN training demands a large dataset and substantial memory, training is ideally only performed one time, then the trained model can infer effectively on new datasets.

This work is an expansion of our associated conference paper [71], containing three primary contributions. First, we detail the design parameters of our CNN and train the novel architecture using simulated and phantom datasets. Second, we classify strain images generated with different SOUL regularization parameter combinations as either suitable or unsuitable using the CNN architecture. Third, we automate the selection of the optimal strain image along with its corresponding regularization parameter set, demonstrating AutoSOUL predictions that enhance the efficiency and reliability of SOUL in ultrasound elastography with multiple test cases, including an in vivo breast lesion example.

The remainder of this article is organized as follows. Section II outlines the SOUL backbone and problem formulation motivating AutoSOUL. Section III describes our datasets, image generation and data labeling processes, CNN architecture, training and testing procedures, final parameter selection and verification steps, and associated performance assessment methods. Section IV details our results. Section V discusses our results, including AutoSOUL benefits, limitations, and potential future work. Finally, Section VI summarizes the key insights obtained from our work.

## II. Problem Statement

Consider two ultrasound beamformed RF frames, represented as $I_{1}(i,j)$ and $I_{2}(i,j)$, captured from a tissue undergoing deformation. In this context, the indices i and j, where 1≤i≤m and 1≤j≤n, correspond to the axial and lateral positions, respectively. Dynamic programming [64] generates the initial axial and lateral displacement maps denoted as $a_{i,j}$ and $l_{i,j}$, respectively. SOUL [66] formulates the following cost function C(⋅) and analytically optimizes [66] this cost function to obtain the incremental displacement fields $\Delta a_{i,j}$ and $\Delta l_{i,j}$ (1), as shown at the top of the page,

(1) $$C\left(\Delta a_{1,1},\ldots ,\Delta a_{m,n},\Delta l_{1,1},\ldots ,\Delta l_{m,n}\right)=\sum_{j=1}^{n}\sum_{i=1}^{m}\left\{D\left(i,j,a_{i,j},l_{i,j},\Delta a_{i,j},\Delta l_{i,j}\right)\right\}+\alpha_{1}{\left\|\partial_{y}a\right\|}_{2}^{2}+\alpha_{2}{\left\|\partial_{x}a\right\|}_{2}^{2}+\beta_{1}{\left\|\partial_{y}l\right\|}_{2}^{2}+\beta_{2}{\left\|\partial_{x}l\right\|}_{2}^{2}+w\alpha_{1}{\left\|\partial_{y}^{2}a\right\|}_{2}^{2}+w\alpha_{2}{\left\|\partial_{x}^{2}a\right\|}_{2}^{2}+w\beta_{1}{\left\|\partial_{y}^{2}l\right\|}_{2}^{2}+w\beta_{2}{\left\|\partial_{x}^{2}l\right\|}_{2}^{2}$$

where $\partial_{y}(\cdot )$ and $\partial_{x}(\cdot )$ refer to first-order axial and lateral derivatives, respectively, $\partial_{y}^{2}(\cdot )$ and $\partial_{x}^{2}(\cdot )$ denote second-order axial and lateral derivatives, respectively, $\alpha_{1}$ and $\alpha_{2}$ regularize the axial displacement field in the axial and lateral directions, respectively, $\beta_{1}$ and $\beta_{2}$ regularize the lateral displacement field in the axial and lateral directions, respectively, w is the ratio between the second-order and first-order regularization weights, and D(⋅) is a measure of data amplitude similarity, defined as follows:

(2) $$D\left(i,j,a_{i,j},l_{i,j},\Delta a_{i,j},\Delta l_{i,j}\right)={\left[I_{1}(i,j)-I_{2}\left(i+a_{i,j}+\Delta a_{i,j},j+l_{i,j}+\Delta l_{i,j}\right)\right]}^{2}.$$

The incremental displacement fields ($\Delta a_{i,j}$ and $\Delta l_{i,j}$) are added to the initial displacements ($a_{i,j}$ and $l_{i,j}$) to calculate the final axial and lateral displacement fields. The final axial displacement is spatially differentiated in the axial direction to obtain the axial strain image.

As axial strain is most frequently utilized in strain elastography applications [5], [33], the focus of this work is to optimize axial strain images, considering that SOUL is not suitable for high-quality lateral displacement/strain estimation [5]. In addition, axial strain image quality is not sensitive to lateral displacement-related parameters (i.e., $\beta_{1}$ and $\beta_{2}$) due to the structure of our cost function formulation. Therefore, this work sets $\beta_{1}$ and $\beta_{2}$ to two nominal values ($\alpha_{1}/2$ and $\alpha_{2}/2$, respectively) to reduce the number of tunable parameters. Based on our observations and experience, SOUL typically demonstrates low sensitivity to the ratio between second-order and first-order regularization weights. Consequently, this study assigns a nominal value of w=100 to maintain consistency without significantly impacting performance. These simplifying actions reduce the number of tunable parameters of SOUL to two: $\alpha_{1}$ and $\alpha_{2}$. Manual tuning of $\alpha_{1}$ and $\alpha_{2}$ makes the displacement/strain calculation process inefficient and user-dependent. Our research problem is to automatically obtain the optimal values of $\alpha_{1}$ and $\alpha_{2}$ for a given dataset. As the SOUL [66] algorithm is inherently robust to variations in transducer center frequency, no compensation is required to account for transmit frequency variations among acquired data.

## III. Methods

### A. Datasets

#### 1) Simulated Data:

A total of 14 simulated phantoms [72], publicly available at data.sonography.ai, were employed (including 10 with one hard inclusion and 4 with two hard inclusions). The background and target elastic moduli were set to 20 and 40 kPa, respectively. The Poisson’s ratio of each simulated phantom was set to 0.49. Each simulated phantom model was deformed with a uniaxial compression level of 2% using the finite element software ABAQUS (Providence, RI). Corresponding pre and postcompression beamformed RF data were simulated with the ultrasound simulation software Field II [73], with ultrasound transmit and temporal sampling frequencies of 5 and 50 MHz, respectively, resulting in 14 total RF frame pairs.

#### 2) Public Phantom Data:

Public experimental data consisted of one pre and postcompression beamformed RF frame pair from an experimental phantom dataset [63], [74], [75], publicly available at data.sonography.ai. To acquire the pre and postcompression frames, a CIRS (Norfolk, VA) breast elastography phantom (Model 059) was subjected to an approximately uniaxial compression due to manual compression. The background elastic modulus was 20 ± 5 kPa. The pre and postcompression beamformed RF frames were acquired using an Alpinion E-Cube 12R research ultrasound scanner equipped with an L3–12H linear array probe. The imaging field of view contained a hard inclusion with an elastic modulus at least twice that of the background. The transmit and sampling frequencies were 10 and 40 MHz, respectively.

#### 3) JHU Phantom Data:

At Johns Hopkins University, five beamformed RF data acquisitions were obtained from a CIRS (Norfolk, VA) breast elastography phantom (Model 059) using an Alpinion E-Cube 12R research ultrasound system with a 128-element L3–8 linear array probe. The imaging field of view for each acquisition contained 1–2 inclusions. The transmit center frequency, sampling frequency, and transmit focus were set to 8 MHz, 40 MHz, and 2 cm, respectively. The background elasticity of the phantom was 20 ± 5 kPa, with inclusions at least twice the elasticity of the background. Two consecutive frames of beamformed RF data were utilized per acquisition.

#### 4) In Vivo Breast Data:

A previously published in vivo dataset [13] from a patient scheduled for an ultrasound-guided core needle biopsy of a suspicious breast mass reported in prior work [76], [77] was imaged prior to biopsy, following informed consent and approval from the Johns Hopkins Medicine Institutional Review Board (Protocol No. IRB00127110). With the patient in the supine position, the breast and mass were axially compressed with a 128-element handheld linear array probe to acquire in vivo raw RF channel data using an Alpinion E-Cube 12R research ultrasound scanner. Two consecutive (pre and postcompression) frames were delay-and-sum beamformed offline. The system operated at a 40-MHz sampling frequency, a 12-MHz transmit center frequency, a 1.2-cm transmit focus, and a 64-element receive aperture. Pathology confirmed the imaged mass to be benign (i.e., stromal fibrosis with focal fibroadenomatoid changes).

### B. Strain Image Generation

**Fig. 1.**
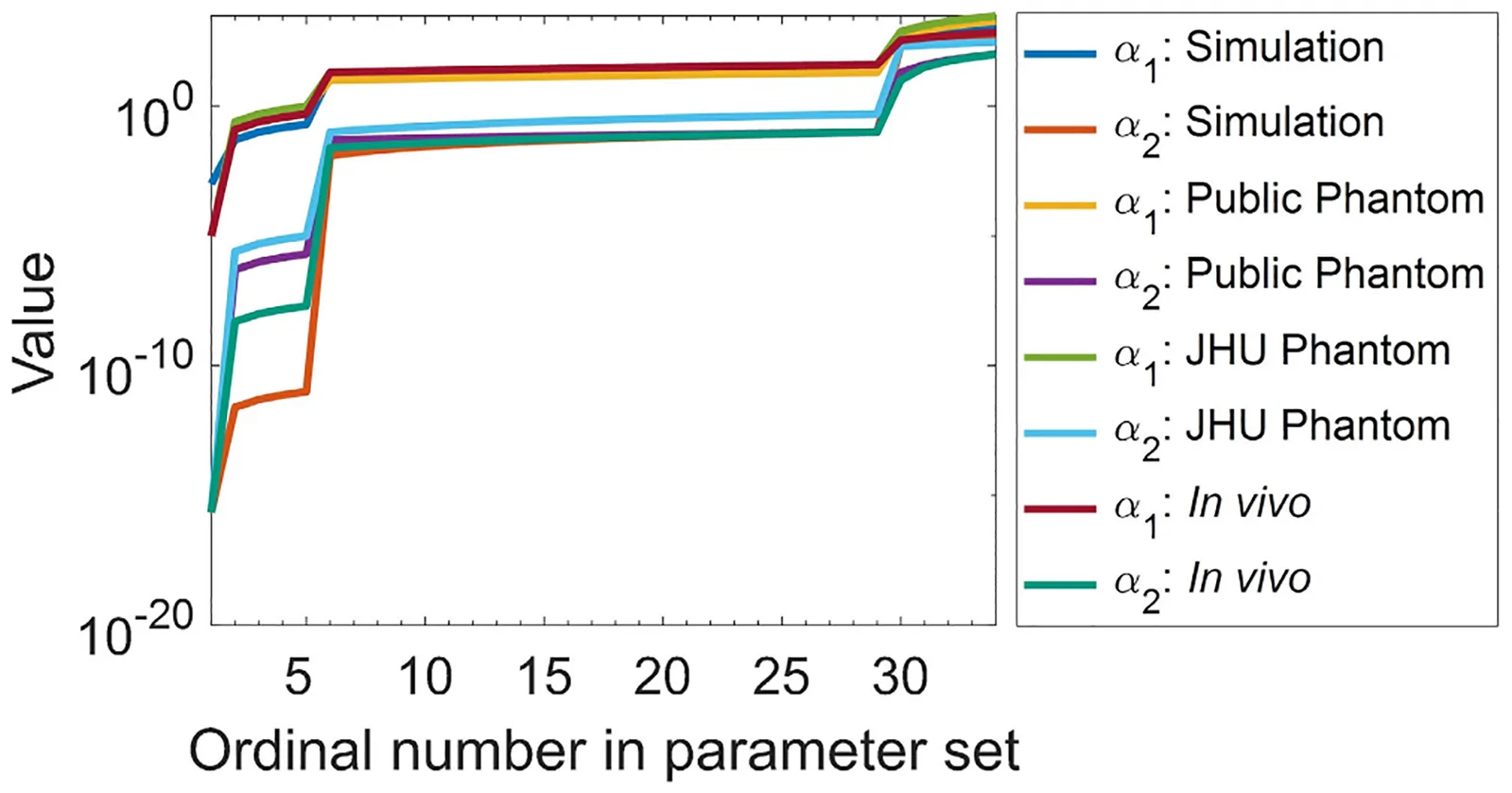
Demonstration of the nonlinear increments within the $\alpha_{1}$ and $\alpha_{2}$ ranges (reported in Table I) per dataset, plotted on a log scale, as a function of the ordinal number when sorted from minimum to maximum $\alpha_{1}$ and $\alpha_{2}$ values per dataset.

We utilized SOUL with a range of $\alpha_{1}$ and $\alpha_{2}$ values (reported in Table I) to estimate displacement fields between 21 (14 simulated, 1 public phantom, 5 JHU phantom, and 1 in vivo breast mass) pre and postcompression beamformed RF frame pairs. The sampling of $\alpha_{1}$ and $\alpha_{2}$ was performed nonlinearly to ensure a wide range of image quality variations (i.e., 5 low values, 5 high values, 24 mid-range values), with the parameter increments for each dataset shown in Fig. 1. The parameters w=100 and $\beta_{1}=\alpha_{1}/2$, or $\beta_{2}=\alpha_{2}/2$ were nominally fixed and scaled, respectively, for the reasons outlined in Section II, which is also supported by the minimal impact observed when varying these parameters during post hoc testing (i.e., after the CNN was trained without the variation). Each axial displacement image was spatially differentiated in the axial direction using a least squares technique [13], [17] to generate the axial strain image. A total of 11595, 1156, 5074, and 132 axial strain images were generated corresponding to simulated, public phantom, JHU phantom, and in vivo breast mass datasets, respectively, as reported in Table I.

### C. Data Labeling for Training and Testing

Two regions of interest (ROIs) of size 50 pixels × 50 pixels (one in the target and the other in the background) were used to calculate the signal-to-noise ratio (SNR) and the strain ratio (SR), which are two commonly used metrics to asses strain image quality. SNR and SR are defined as follows:

(3) $$\text{SNR}=\frac{{\overset{‾}{s}}_{b}}{\sigma_{b}}$$

(4) $$\text{SR}=\frac{{\overset{‾}{s}}_{t}}{{\overset{‾}{s}}_{b}}$$

where ${\overset{‾}{s}}_{b}$ and ${\overset{‾}{s}}_{t}$ refer to the mean strain values calculated within the background and target ROIs, respectively, and $\sigma_{b}$ is the standard deviation of the strain values in the background ROI. The background region was used for SNR computation, as it is conventionally adopted in ultrasound elastography studies to quantify uniformity and noise characteristics [65].

**Fig. 2.**
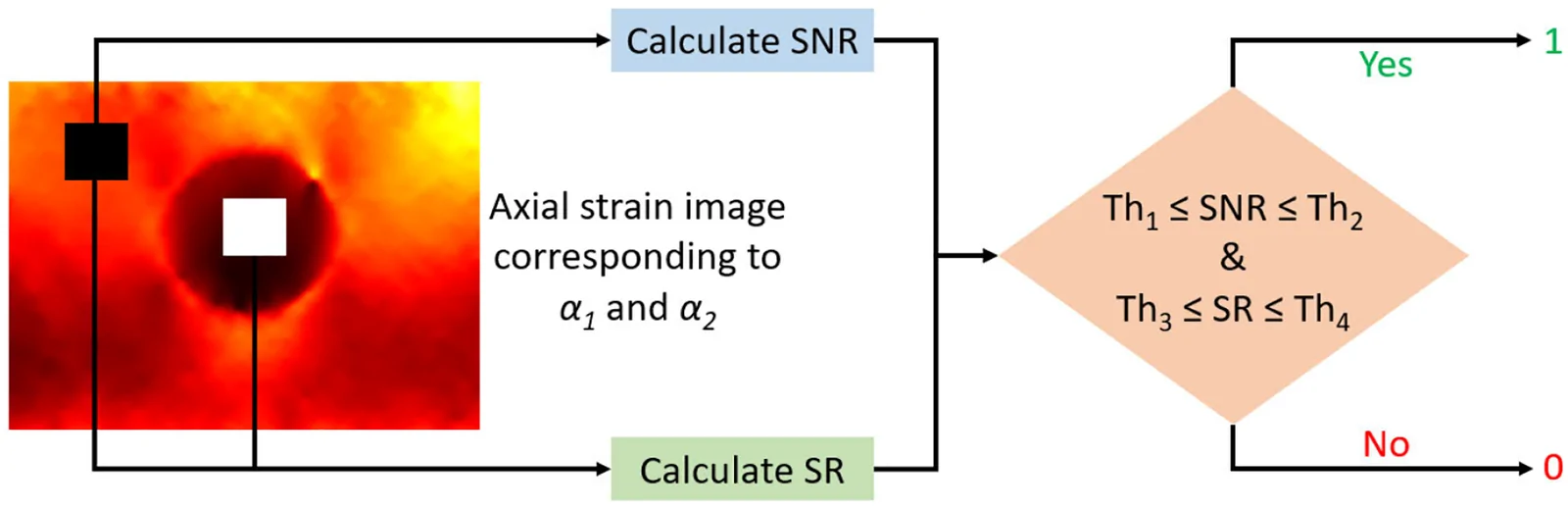
flowchart summarizing the strain image labeling process. Th_1_ and Th_2_ denote the minimum and maximum thresholds for the acceptable SNR values, whereas Th_3_ and Th_4_ refer to the minimum and maximum thresholds for the acceptable SR values.

Background smoothness, target-to-background contrast, and lesion boundary sharpness were qualitatively assessed to establish acceptable SNR and SR ranges for strain images from each pre and postcompression frame pair. These ranges determined whether or not strain images were labeled as acceptable (1) or unacceptable (0), as illustrated in Fig. 2. Acceptable SNR values ranged 15–120, 22–45, 10–90, and 15–20 for simulated, public phantom, JHU phantom, and in vivo breast mass datasets, respectively, whereas acceptable SR values ranged 0.5–0.68, 0.39–0.42, 0.22–0.5, and 0.55–0.63 for simulated, public phantom, JHU phantom, and in vivo breast mass datasets, respectively. There is an upper bound on the SNR values because we observed that SNRs that are too high (e.g., > 120) tend to be associated with oversmoothed backgrounds, blurred lesion boundaries, and reduced contrast between the target and background. This rationale was similarly applied to determine lower bounds and SR bounds.

For training, 9484 (3459 acceptable, 6025 unacceptable) and 3917 (1957 acceptable, 1960 unacceptable) unique strain images from 12 simulated and 4 JHU phantom frame pairs, respectively, were used. Testing utilized 955 (375 acceptable, 580 unacceptable), 1156 (576 acceptable, 580 unacceptable), 1156 (576 acceptable, 580 unacceptable), 1157 (597 acceptable, 560 unacceptable), and 132 (66 acceptable, 66 unacceptable) strain images from one RF frame pair test case per single-inclusion simulated, multi-inclusion simulated, public phantom, JHU phantom (multi-inclusion), and in vivo breast mass dataset, respectively. The total number of unique acceptable and unacceptable strain images are summarized in Table I. Strain image generation and data labeling were performed with MATLAB software (The MathWorks, Inc., Natick, MA).

Strain images were normalized between 0 and 1 and resized to 512 × 512 pixels in preparation for training and testing. After augmenting with laterally flipped duplicates, the total number of JHU phantom training images increased to 7834 (3914 acceptable, 3920 unacceptable). Only the JHU phantom training data was augmented due to its smaller number of training examples and limited diversity of inclusion locations compared to the simulated training data. The augmented training data are not included in the unique label and image counts reported in Table I.

### D. CNN Architecture

**Fig. 3.**
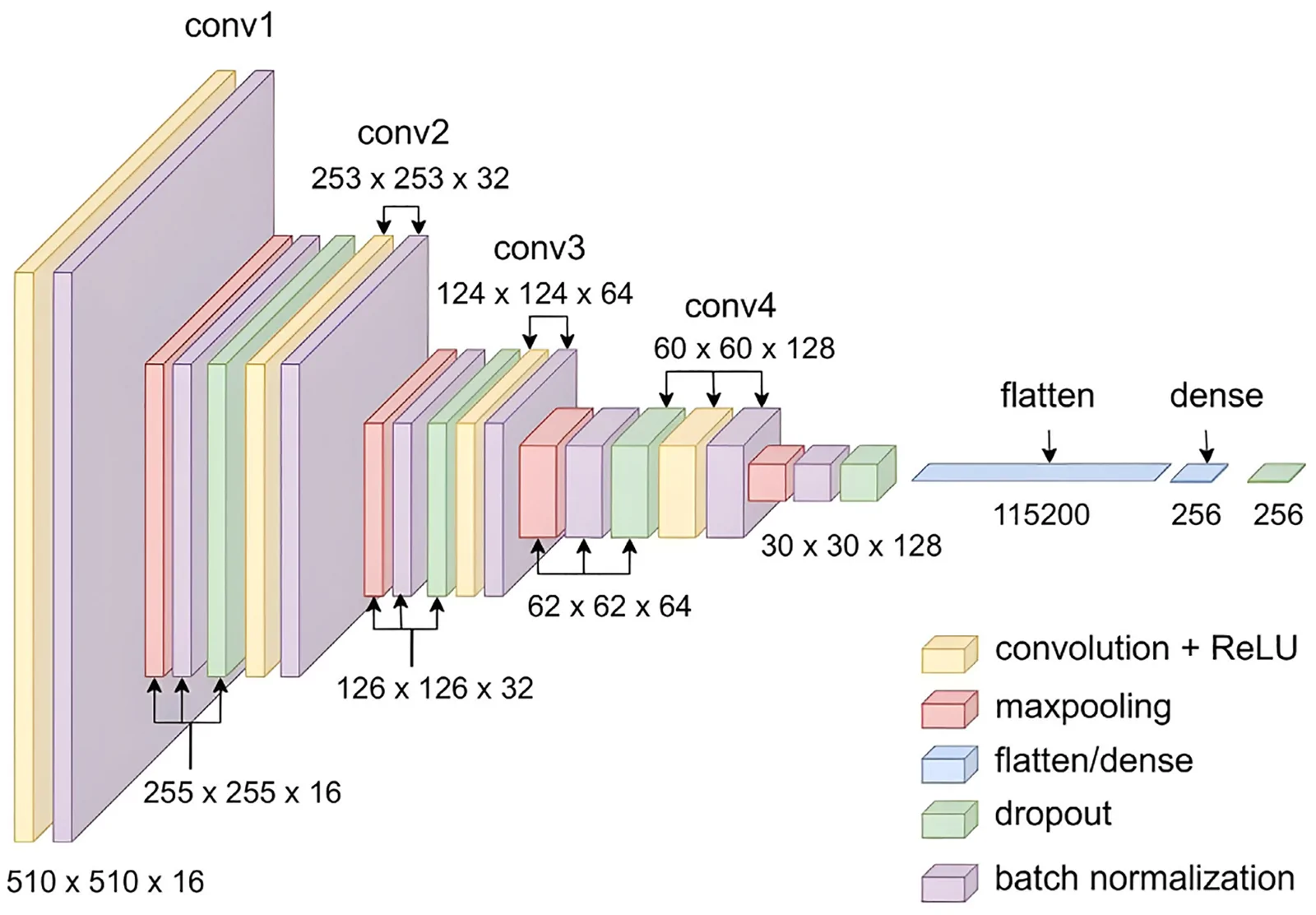
CNN architecture employed in AutoSOUL.

We designed a binary classification CNN to evaluate input strain images and produce a probability score between 0 and 1, where outputs greater than 0.5 were classified as acceptable. The network comprised four 2-D convolutional layers (3 × 3 kernel, stride 1, ReLU activation), each followed by max-pooling and 30% dropout layers. The convolutional layers used 16, 32, 64, and 128 filters, respectively. The 3-D feature maps were flattened into 1-D vectors and passed through a fully connected layer with 256 units and ReLU activation, followed by a 50% dropout to mitigate overfitting. Finally, a fully connected output layer with a single unit and sigmoid activation performed the binary classification of strain images. Fig. 3 illustrates the CNN architecture.

### E. Training, Fine-Tuning, and Testing of the CNN

The CNN was initially trained on simulated images (80%–20% training–validation split) using a binary cross-entropy loss function. The Adam optimizer, with a learning rate of 0.001 and a batch size of 32, was employed. Training was conducted for 200 epochs, with early stopping applied to prevent overfitting based on validation loss.

To enhance generalizability, the pretrained CNN was fine-tuned on phantom training images (80%–20% training–validation split). Fine-tuning was performed using the binary cross-entropy loss function for up to 200 epochs, with early stopping to mitigate overfitting. The Adam optimizer was used with a reduced learning rate of 3 × 10^−5^ and a batch size of 32. Training and fine-tuning were implemented using Keras on a NVIDIA Quadro RTX 6000 GPU with 24 GB GPU memory.

The trained and fine-tuned CNN was tested on the single-inclusion simulated, multi-inclusion simulated, public phantom, JHU phantom, and in vivo breast test datasets described in Section III-C. For each tested strain image, an output probability prediction score > 0.5 was considered acceptable. This prediction does not require images containing SR and SNR values that were included during the training process, whether inside or outside of the range of acceptable SR and SNR values used during the data labeling process.

### F. CNN Performance Assessments

To evaluate the performance of the CNN to classify strain images as acceptable or unacceptable, we measured accuracy, precision, recall, and F1-scores (four widely recognized classification metrics), which are defined below

(5) $$\text{Accuracy}=\frac{\text{TP}+\text{TN}}{\text{TP}+\text{TN}+\text{FP}+\text{FN}}$$

(6) $$\text{Precision}=\frac{\text{TP}}{\text{TP}+\text{FP}}$$

(7) $$\text{Recall}=\frac{\text{TP}}{\text{TP}+\text{FN}}$$

(8) $$F1\;\text{Score}=2\cdot \frac{\text{Precision}\cdot \text{Recall}}{\text{Precision}+\text{Recall}}$$

where TP corresponds to an image that is correctly classified as acceptable (i.e., true positive), FP corresponds to an image incorrectly classified as acceptable (i.e., false positive), TN corresponds to an image correctly classified as unacceptable (i.e., true negative), and FN corresponds to an image incorrectly classified as unacceptable (i.e., false negative). Accuracy measures the proportion of correctly classified instances out of the total instances. Precision quantifies the ratio of true positive predictions to the total predicted positives, while recall represents the ratio of true positives to the total actual positives. The F1-score provides a balanced measure of the performance, particularly in scenarios with imbalanced class distributions. These metrics collectively provide a comprehensive assessment of the classification efficacy of our CNN.

### G. Optimal Parameter Set Selection

**Fig. 4.**
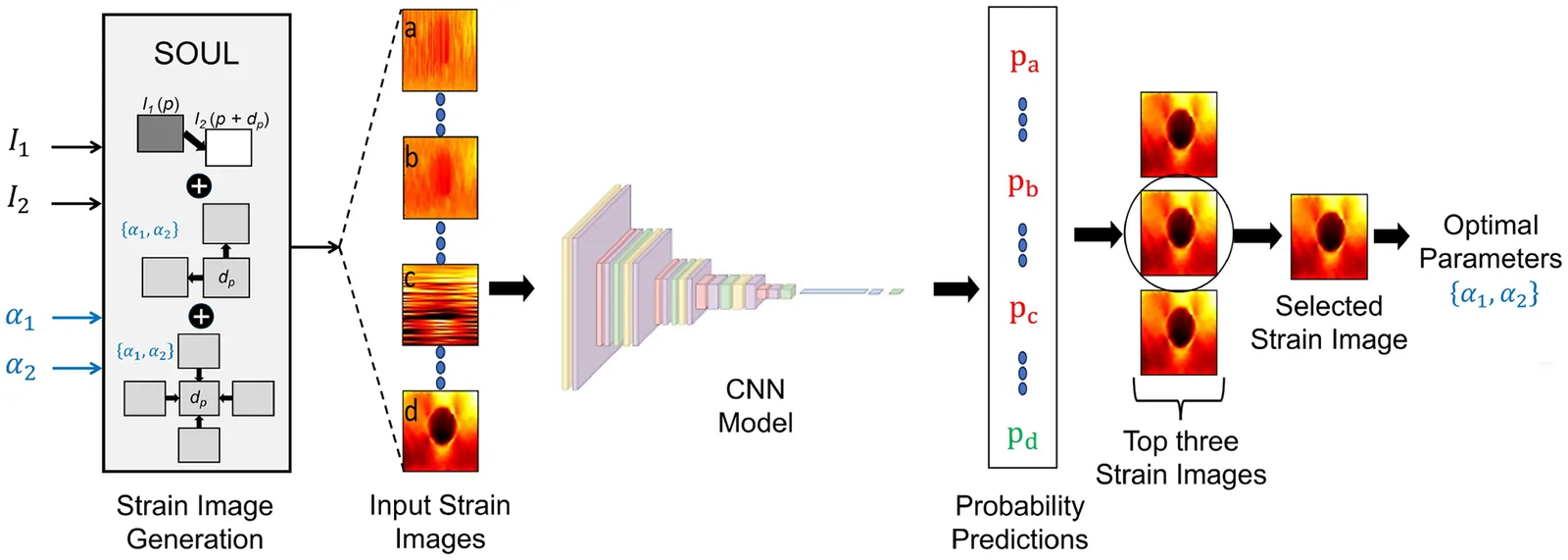
Summary of the AutoSOUL framework.

To select the optimal SOUL parameters and ensure the absence of false positives (i.e., an unacceptable strain image considered acceptable by the CNN), the strain images corresponding to the top three probabilities predicted by the CNN for a given pre and postcompression RF frame set were visually inspected. To ensure the final selection of an “acceptable” image, the SOUL regularization parameter set (i.e., $\alpha_{1}$ and $\alpha_{2}$) associated with the strain image that qualitatively balanced background smoothness, target-to-background contrast, and boundary sharpness was considered the optimal parameter set. The entire process from CNN input to final parameter selection, including the automatic SOUL parameter selection process, is named AutoSOUL and is illustrated in Fig. 4.

### H. AutoSOUL Versus SOUL Performance Assessments

To benchmark AutoSOUL performance, we visually inspected strain images from the testing dataset, selecting one image per test case that best balanced three key visual features: background smoothness, target-to-background contrast, and target boundary sharpness. The $\alpha_{1}$ and $\alpha_{2}$ values of each selected image were considered the manually tuned optimal parameter sets. This type of manual parameter tuning is standard with SOUL. To assess the similarity between SOUL- and AutoSOUL-determined parameters, parameter ratios were quantified (i.e., ${\alpha_{1}}^{\text{AutoSOUL}}/\alpha_{1}^{\text{SOUL}}$ and ${\alpha_{2}}^{\text{AutoSOUL}}/\alpha_{2}^{\text{SOUL}}$) per test case.

To assess the objectivity of the AutoSOUL parameter selection process, strain images produced by AutoSOUL were compared to the traditional SOUL results across the five test cases (i.e., single-inclusion simulation, multi-inclusion simulation, public phantom, JHU phantom, and in vivo breast mass) initially with qualitative evaluations. In addition to qualitative evaluations, the root-mean-square error (RMSE) and mean absolute error (MAE) between the estimated and ground truth strain images were provided as strain estimation accuracy measurement metrics, defined as follows:

(9) $$\text{RMSE}=\sqrt{\frac{\sum_{j=1}^{n}\sum_{i=1}^{m}{\left({\overset{ˆ}{s}}_{i,j}-s_{i,j}\right)}^{2}}{\text{mn}}}$$

(10) $$\text{MAE}=\frac{\sum_{j=1}^{n}\sum_{i=1}^{m}\left|{\overset{ˆ}{s}}_{i,j}-s_{i,j}\right|}{\text{mn}}$$

where ${\overset{ˆ}{s}}_{i,j}$ and $s_{i,j}$ are the estimated and ground truth strains, respectively, for the pixel values located at index (i,j), with m and n total samples, respectively.

Elastographic contrast-to-noise ratio (CNR) [3], SNR [3], [78], and SR were additionally included as image quality assessment metrics, with SNR and SR defined in Section III-C and CNR defined as follows:

(11) $$\text{CNR}=\sqrt{\frac{2{\left({\overset{‾}{s}}_{b}-{\overset{‾}{s}}_{t}\right)}^{2}}{\sigma_{b}^{2}+\sigma_{t}^{2}}}$$

where $\sigma_{t}$ is the standard deviation of strain values within the target ROI.

Rather than report a single SNR, CNR, or SR value per dataset, as is commonly done in previous elastography performance studies [15], each dataset was evaluated using 30 SNR background ROIs (each measuring 3 × 3 mm) and 120 CNR and SR ROI combinations [13]. These combinations consisted of four 3 × 3 mm target ROIs paired with the same 30 background ROIs used for SNR calculations, providing a more comprehensive assessment of overall image quality. ROIs were selected to contain visually uniform background and target strains. The lateral locations of the background ROI centers ranged 7.59–26.15 mm (single-inclusion simulation), 6.86–25.52 mm (multi-inclusion simulation), 7.78–31.71 mm (public phantom), 8.14–25.36 mm (JHU phantom), and 4.25–21.41 mm (in vivo). The axial locations of the background ROI centers ranged 9.63–33.03 mm (simulations), 5.40–26.57 mm (public phantom), 10.43–30.64 mm (JHU phantom), and 7.53–16.78 mm (in vivo).

Execution times between SOUL and AutoSOUL were compared using a total of 1000 strain images. In particular, the manual inspection of 1000 strain images required by SOUL during exhaustive parameter search (performed by coauthor MJA) was contrasted with the automated selection process performed by AutoSOUL. The performance time reduction from SOUL to AutoSOUL was measured. The AutoSOUL process was performed using a workstation equipped with an Intel^1^ Core^2^ i7-12700K CPU (3.60 GHz) and an NVIDIA Quadro RTX 6000 GPU.

## IV. Results

### A. Probability Predictions and Associated Parameters

**Fig. 5.**
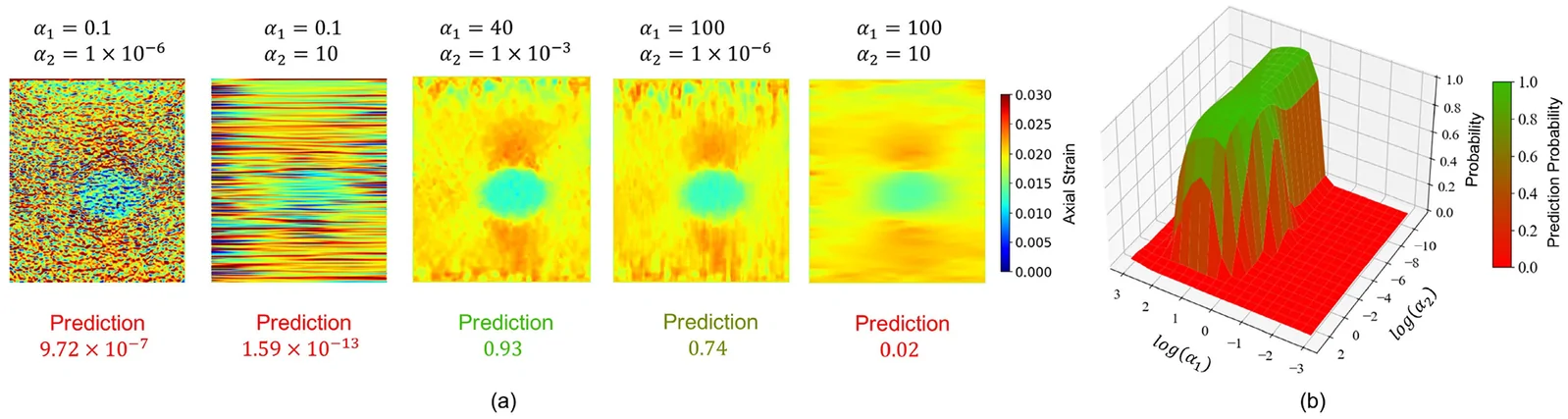
(a) Example of strain images from the single-inclusion simulation test case, obtained with varying $\alpha_{1}$ and $\alpha_{2}$ parameter combinations and corresponding CNN prediction probabilities. (b) CNN prediction probability distribution from the single-inclusion simulation test case as a function of $\text{log-}\alpha_{1}$ and $\text{log-}\alpha_{2}$ values.

Fig. 5 shows representative strain images generated using different combinations of $\alpha_{1}$ and $\alpha_{2}$, corresponding prediction probabilities, and the distribution of CNN prediction probabilities as a function of $\alpha_{1}$ and $\alpha_{2}$, using the single-inclusion simulation test case. In Fig. 5(a), examples are shown to demonstrate that the CNN predicts low to near-zero probabilities for noisy, streaked, and blurred images with indistinct lesion boundaries. In contrast, parameter settings that produce strain images with smooth backgrounds and sharp lesion boundaries achieve high probabilities (i.e., 0.93). Intermediate cases (e.g., $\alpha_{1}=100,\alpha_{2}=10^{-6}$) show moderate probabilities (i.e., 0.74), consistent with acceptable but not optimal performance. In Fig. 5(b), a concentrated plateau is observed with high prediction probability region (i.e., >0.9), where the corresponding parameters produce stable predictions with minimal variation. In contrast, low-probability regions (i.e., near 0) are widely distributed across $\alpha_{1}$ and $\alpha_{2}$ values, corresponding to parameter combinations associated with unacceptable strain images. Similar features were obtained with the remaining test cases.

### B. Classification Performance

Table II reports the classification performance of the proposed CNN on single-inclusion simulated, multi-inclusion simulated, public phantom, JHU phantom, and the in vivo breast mass datasets. The classification metric values for the simulated datasets range from 89.09% (precision for the multi-inclusion simulated data) to 100% (recall for single-inclusion and multi-inclusion simulated datasets). Experimental phantom datasets generally have greater classification metric values than the simulated datasets, ranging from 96.62% (recall for JHU phantom dataset) to 100% (recall for public phantom and precision for JHU phantom datasets). This better performance in experimental data relative to simulated data is likely due to fine-tuning of the classifier on experimental phantom strain images. Lower classification performance was observed with the in vivo breast mass dataset (i.e., metric values ranging from 68.1% precision to 100% recall), likely due to a combination of the domain shift between simulated or phantom data and in vivo data and the absence of in vivo training data. Overall, the F1-scores exceed 81%, which is an acceptable performance balance among the reported metrics for each dataset.

### C. Strain Imaging Performance

#### 1) Single-Inclusion Simulated Test Data:

**Fig. 6.**
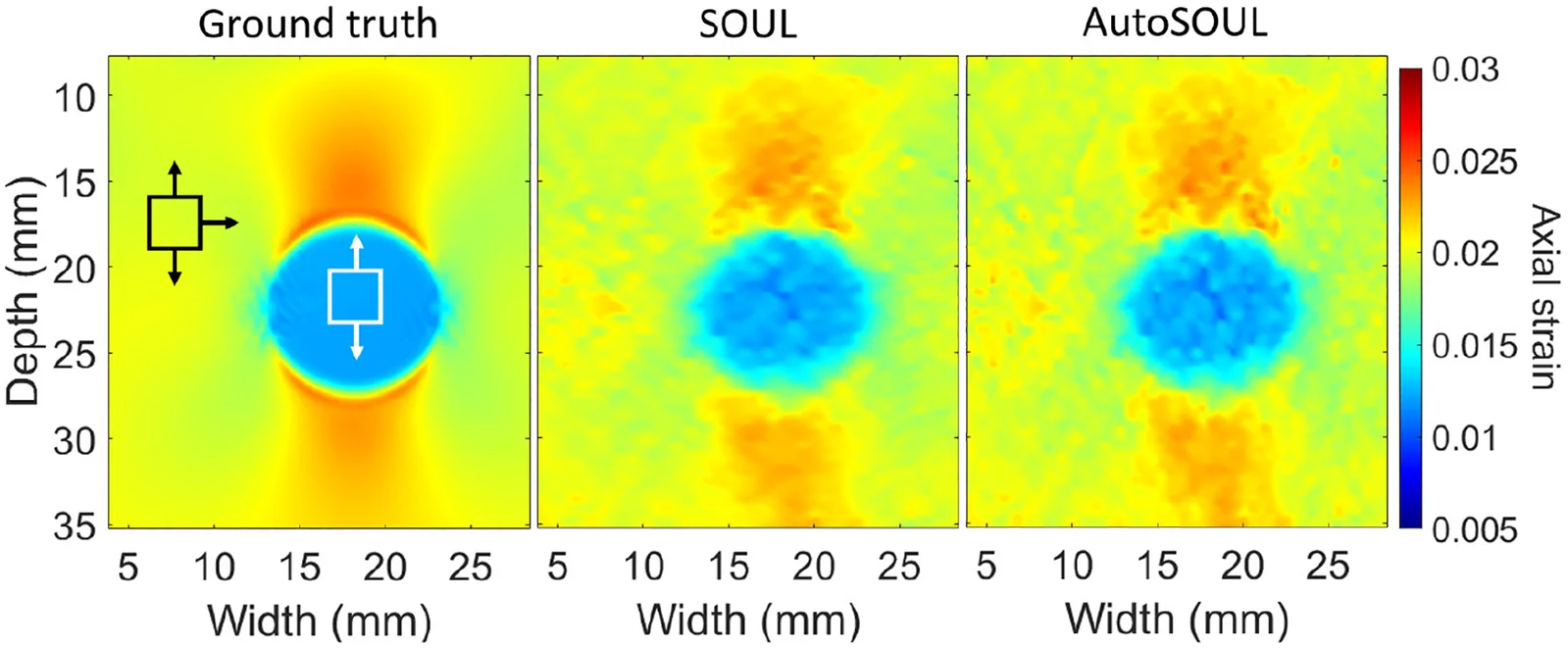
Axial strain results obtained with the single-inclusion simulated dataset. The white and black boxes indicate target and background ROIs, respectively, utilized to calculate one of the 30 SNR measurements (black ROI only), one of the 120 CNR, and one of the 120 SR measurements. The arrows indicate the directions of ROI displacement to obtain the remaining SNR, CNR, and SR values.

Fig. 6 shows the ground truth axial strain image and the axial strain images obtained by SOUL $\left(\alpha_{1}=40,\alpha_{2}=0.05\right)$ and the proposed automatic technique AutoSOUL $\left(\alpha_{1}=38.26,\alpha_{2}=0.0055\right)$ for the single-inclusion simulated dataset (i.e., ${\alpha_{1}}^{\text{AutoSOUL}}/\alpha_{1}^{\text{SOUL}}=0.96$ and ${\alpha_{2}}^{\text{AutoSOUL}}/\alpha_{2}^{\text{SOUL}}=0.11$). Both SOUL and AutoSOUL strain images resemble the ground truth and reveal the hard inclusion. The SOUL strain image is visually smoother than the AutoSOUL strain image. However, the AutoSOUL strain image is comparable to the SOUL counterpart in terms of sharpness of the inclusion boundary and the visual contrast between the target and background, as supported by the corresponding values reported in Table III. RMSE and MAE values corresponding to the AutoSOUL strain image are minimally greater than those corresponding to the SOUL strain image (i.e., by 4.05% and 9.17%, respectively). The first row of image quality metrics in Table IV demonstrates that the AutoSOUL strain image renders 15.01% and 12.69% lower SNR and CNR, respectively, when compared to the SOUL image, with identical SR values.

#### 2) Multi-Inclusion Simulated Test Data:

**Fig. 7.**
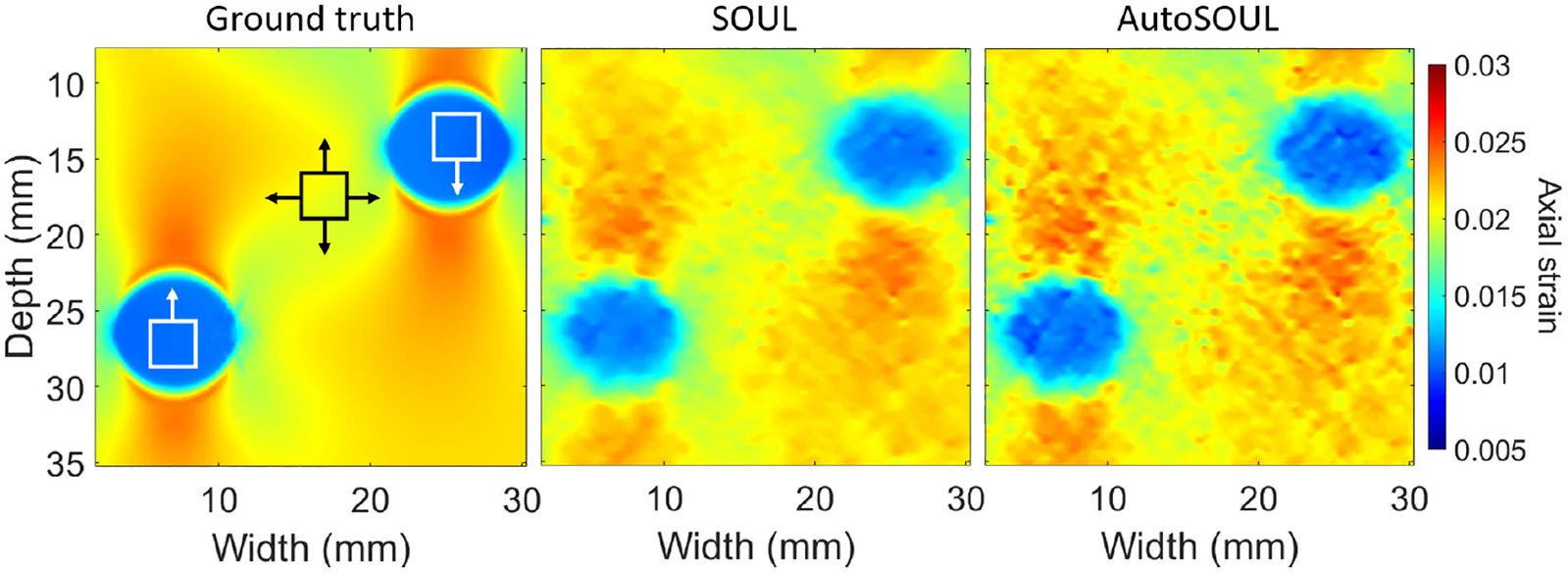
Axial strain results obtained with the multi-inclusion simulated dataset. The white and black boxes indicate two target ROIs and one background ROI, respectively, utilized to calculate one of the 30 SNR measurements (black ROI only), two of the 120 CNR, and two of the 120 SR measurements. The arrows indicate the directions of ROI displacement to obtain the remaining SNR and CNR values.

Fig. 7 shows the ground truth strain image along with the SOUL $\left(\alpha_{1}=40,\alpha_{2}=0.05\right)$ and AutoSOUL $\left(\alpha_{1}=19.35,\alpha_{2}=0.0125\right)$ strain images for the multi-inclusion simulated dataset (i.e., ${\alpha_{1}}^{\text{AutoSOUL}}/\alpha_{1}^{\text{SOUL}}=0.48$ and ${\alpha_{2}}^{\text{AutoSOUL}}/\alpha_{2}^{\text{SOUL}}=0.25$). Both the SOUL and AutoSOUL strain images closely match the ground truth and display the two stiff inclusions. Although the SOUL strain image presents a visually smoother background, the AutoSOUL strain image produces sharper inclusion boundaries and greater visual contrast between the background and the stiff inclusions. The quantitative values in Tables III and IV are consistent with our qualitative observations. In particular, the AutoSOUL strain image has 1.5% lower (i.e., better) RMSE, 6.49% higher MAE, 30.11% lower SNR, 21.04% lower CNR, and 3.70% lower (i.e., improved) SR values.

#### 3) Public Phantom Test Data:

**Fig. 8.**
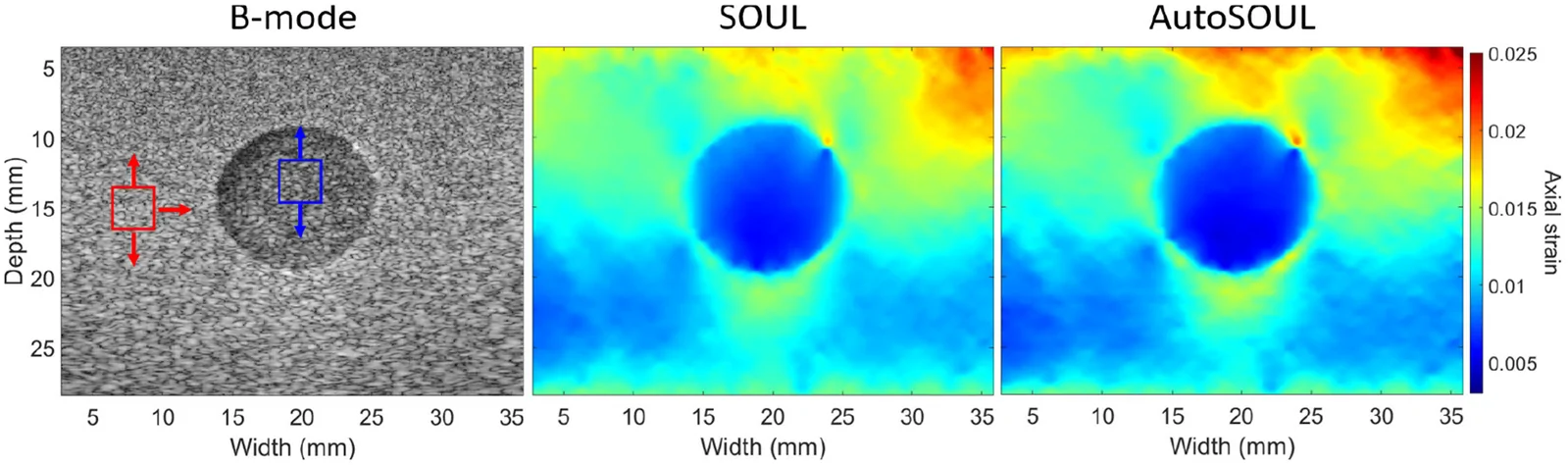
Axial strain results obtained with the public phantom dataset. The B-mode image is shown with 40-dB dynamic range. The blue and red boxes indicate target and background ROIs, respectively, utilized to calculate one of the 30 SNR measurements (blue ROI only), one of the 120 CNR, and one of the 120 SR measurements. The arrows indicate the directions of ROI displacement to obtain the remaining SNR, CNR, and SR values.

Fig. 8 shows the B-mode image alongside the SOUL $\left(\alpha_{1}=40,\alpha_{2}=0.05\right)$ and AutoSOUL $\left(\alpha_{1}=20,\alpha_{2}=0.05\right)$ strain images for the public phantom dataset (i.e., ${\alpha_{1}}^{\text{AutoSOUL}}/\alpha_{1}^{\text{SOUL}}=0.5$ and ${\alpha_{2}}^{\text{AutoSOUL}}/\alpha_{2}^{\text{SOUL}}=1.0$). SOUL and AutoSOUL strain images distinctly reveal the hard inclusion. The SOUL strain image has a marginally smoother background, and the AutoSOUL image appears to have lower strain values within the lesion. These observations translate to AutoSOUL strain images with 11.19% lower SNR, 1.66% lower CNR, and 5.66% improved SR, as reported in Table IV.

#### 4) JHU Phantom Test Data:

**Fig. 9.**
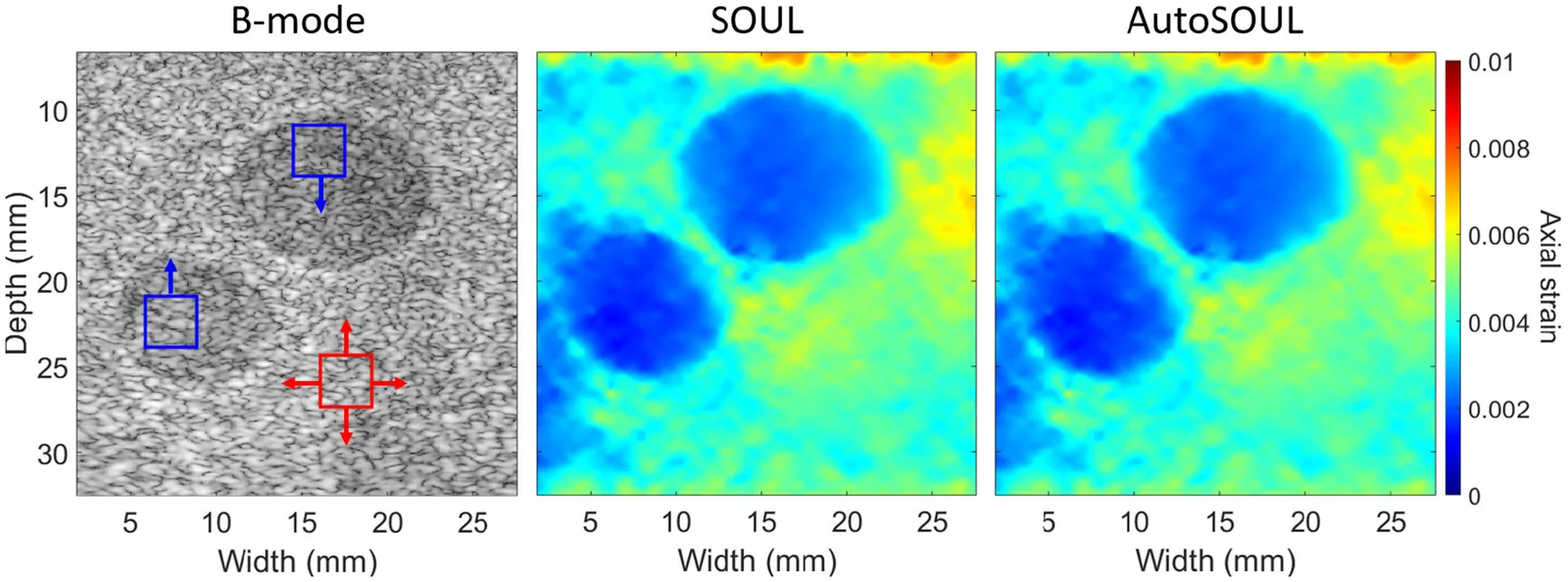
Axial strain results obtained with the JHU phantom dataset. The B-mode image is shown with 40-dB dynamic range. The blue and red boxes indicate target and background ROIs, respectively, utilized to calculate one of the 30 SNR measurements (blue ROI only), two of the 120 CNR, and two of the 120 SR measurements. The arrows indicate the directions of ROI displacement to obtain the remaining SNR, CNR, and SR values.

Fig. 9 shows the B-mode image and the corresponding SOUL $\left(\alpha_{1}=40,\alpha_{2}=0.05\right)$ and AutoSOUL $\left(\alpha_{1}=40,\alpha_{2}=0.05\right)$ strain images for the JHU phantom dataset (i.e., ${\alpha_{1}}^{\text{AutoSOUL}}/\alpha_{1}^{\text{SOUL}}=1$ and ${\alpha_{2}}^{\text{AutoSOUL}}/\alpha_{2}^{\text{SOUL}}=1$). The SOUL and AutoSOUL strain images are identical (and so are the associated SNR, CNR, and SR values reported in Table IV), as these two images were created with the same regularization parameter values. Both images exhibit high quality, with the two hard inclusions distinctly visible against the soft backgrounds.

#### 5) In Vivo Breast Mass Data:

**Fig. 10.**
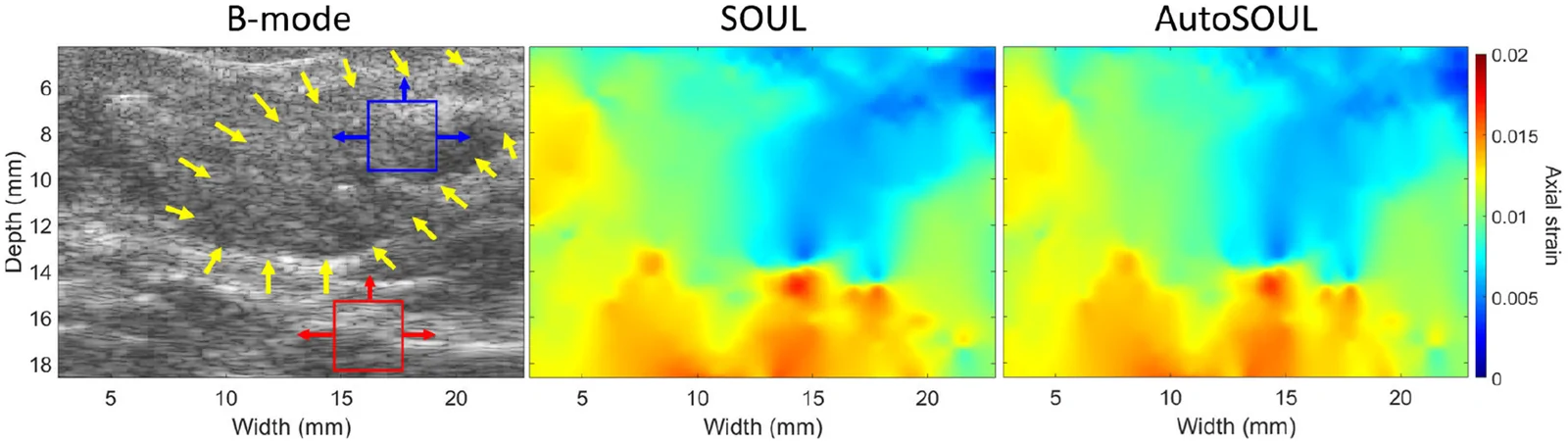
Axial strain results obtained with the in vivo breast mass dataset. The B-mode image is shown with 50-dB dynamic range. Yellow arrows indicate the mass boundary [13]. The blue and red boxes indicate target and background ROIs, respectively, utilized to calculate one of the 30 SNR measurements (blue ROI only), one of the 120 CNR, and one of the 120 SR measurements. The arrows indicate the directions of ROI displacement to obtain the remaining SNR, CNR, and SR values.

Fig. 10 shows the in vivo B-mode image alongside the SOUL $\left(\alpha_{1}=20,\alpha_{2}=0.025\right)$ and AutoSOUL $\left(\alpha_{1}=28,\alpha_{2}=0.0125\right)$ strain images (i.e., ${\alpha_{1}}^{\text{AutoSOUL}}/\alpha_{1}^{\text{SOUL}}=1.4$ and ${\alpha_{2}}^{\text{AutoSOUL}}/\alpha_{2}^{\text{SOUL}}=0.5$). SOUL and AutoSOUL strain images effectively differentiate the breast mass from the surrounding healthy tissue. The AutoSOUL strain image has a marginally smoother background than the SOUL strain image. The quantitative values in Table IV reveal that the AutoSOUL strain image has 12.63%, 0.87%, and 6.12% higher SNR, CNR, and SR values, respectively, relative to the SOUL strain image.

### D. Execution Time

The manual inspection time of 1000 strain images due to an exhaustive parameter search requiring 3 s per image is 3000 s. Twenty images were then shortlisted, with pairwise comparisons totaling an additional 20 choose 2 = 190 comparisons, which adds 570 s to the assessments (i.e., considering the manual inspection time of 3 s per image). Therefore, the total tuning time for SOUL is 3570 s (i.e., approximately 1 h). In comparison, AutoSOUL requires a single forward pass of the CNN model to select the optimal parameters. With a GPU inference time of 33.3 ms per image, evaluating 1000 images requires 33.3 s, plus an additional 9 s (i.e., to perform the top 3 choose 2 = 3 comparisons at a rate of 3 s per comparison) for the final assessment. Therefore, the total execution time with AutoSOUL is 42.3 s, which is a factor of 84.4 reduction relative to the parameter selection execution time for SOUL.

## V. Discussion

This research is the first to propose a deep learning-based framework (i.e., AutoSOUL) to autonomously select the optimal parameter set for a regularized energy-based displacement tracking technique for ultrasound strain elastography. Qualitative (Figs. 6–10) and quantitative (Tables II–IV) results obtained from simulated, phantom, and in vivo datasets validate the potential of AutoSOUL to automatically estimate the optimal elastographic displacement tracking parameters. Qualitatively, the strain images (Figs. 6–10) corresponding to the parameter sets suggested by AutoSOUL are comparable to the manually tuned parameter sets in terms of background smoothness, target-to-background contrast, and the sharpness of the target boundaries. These qualitative assessments are consistent with RMSE and MAE comparisons to known ground truth values (Table III) and with SNR, CNR, and SR image quality metrics (Table IV).

Although CNR was a metric used during benchmarking, it was not used to determine acceptable and unacceptable images during the data labeling process. Therefore, our combination of SNR (which considers background smoothness) and SR (which considers lesion-to-background contrast) during the data labeling process seems to have effectively met our objectives without introducing a third metric that would serve a similar purpose, allowing us to streamline the data labeling process. While alternative metrics could be used for benchmarking and data labeling in future work, it is important to note that the bounded generalized contrast-to-noise ratio (gCNR) [79] was not a suitable metric for our framework, because blurry and smeared background-to-target boundaries had near-unity gCNR. The unbounded metrics of SR and SNR introduced greater flexibility to impose data-specific bounds, as described in Section III-C. It is promising that the AutoSOUL CNN correctly predicted unacceptable strain images under some of the most challenging conditions for other metrics (e.g., gCNR).

The primary benefit of AutoSOUL is the factor of 84.4 reduction in the parameter selection execution time (as reported in Section IV-D). In addition, the associated CNN model produced reliable and stable outputs without explicitly learning $\alpha_{1}$ and $\alpha_{2}$ during training (Fig. 5), resulting in ${\alpha_{1}}^{\text{AutoSOUL}}/\alpha_{1}^{\text{SOUL}}$ and ${\alpha_{2}}^{\text{AutoSOUL}}/\alpha_{2}^{\text{SOUL}}$ ranges of 0.48−1.4 and 0.11−1, respectively (mean: 0.87 and 0.57, respectively), across the five test cases. In one of the five test cases, AutoSOUL produced an identical strain image compared to that produced by SOUL (Fig. 9). The remaining four test cases produced image quality measurements (i.e., SNR, CNR, and SR) that deviated by 0%−30.11%. This range of agreement between the two approaches indicates that the automatic technique can achieve a similar standard of strain image quality relative to the manual process without the need for labor-intensive hand-tuning of parameters. Such consistency underscores the robustness and efficiency of AutoSOUL, making it a promising alternative to strain imaging with completely manual parameter selections.

The in vivo breast mass results (Fig. 10 and Table IV) highlight the performance of AutoSOUL in a clinical context, demonstrating its ability to effectively differentiate a breast mass from surrounding healthy tissue, comparable to the manually tuned strain image. The smoother background of the AutoSOUL image in Fig. 10 is likely due to the higher CNN-suggested $\alpha_{1}$ value, relative to the manually selected SOUL value. This improvement suggests that AutoSOUL not only maintains diagnostic relevance but also has the potential to enhance image quality in some cases. By eliminating the need for manual parameter tuning, the proposed method can streamline workflows and reduce variability, making high-quality elastography more accessible in clinical settings. This advancement paves the way for broader applications in tissue mechanics assessment, eventually contributing to more precise and timely patient care.

The promising results of AutoSOUL across simulated, phantom, and in vivo datasets demonstrate its robustness and potential for broader applications in elastography. By successfully automating axial strain estimation, this approach establishes a framework that can be extended to other strain imaging modalities, such as lateral and shear strain imaging [5], [13], where automatic parameter selection remains a significant challenge. In addition, the underlying principles of this method can potentially be adapted to other elastography techniques, including shear wave elastography [11], to streamline parameter optimization and enhance reproducibility.

One limitation of the presented approach is the 77.31% accuracy and 68.1% precision achieved with the in vivo breast mass data, despite the 81.04% F1-score and 100% recall (Table II). This performance discrepancy relative to the simulated and phantom test cases may stem from the inherent domain shift between the well-controlled simulated or phantom environments and the more complex in vivo tissue. The absence of in vivo data during training likely limited the ability of our CNN to generalize to clinical scenarios.

Potential future work includes enhancing generalizability and robustness or exploring more advanced network architectures to ensure that intricate in vivo features will be considered. In particular, transformer-based [80] or Mamba-based [81] models have shown superior performance in various domains [82], [83], due to their ability to capture long-range dependencies and complex patterns. These options have the potential to improve the accuracy and reliability of parameter selection in strain imaging.

## VI. Conclusion

We proposed AutoSOUL, a new deep learning-based method to select the optimal parameter set for SOUL, which is an energy-based algorithm for speckle tracking in ultrasound strain elastography. A custom-designed CNN was trained on simulated and phantom datasets to classify axial strain images as acceptable or unacceptable and to recommend the optimal SOUL parameter set for a given dataset. In simulated, phantom, and in vivo breast mass experiments, the strain images generated using AutoSOUL closely matched those obtained through exhaustive manual tuning, achieving 0–30.11% difference in SNR, CNR, and SR values. These results demonstrate that AutoSOUL can effectively automate the selection of speckle tracking parameters, reducing user dependence and enhancing the efficiency of ultrasound strain imaging. Ultimately, AutoSOUL has the potential to streamline elastography-guided disease diagnosis, treatment monitoring, and clinical intervention.

## Figures and Tables

**TABLE I:** Parameter Ranges and Distribution of Images With Acceptable and Unacceptable Labels for AutoSOUL Training/Testing

Dataset	$\alpha_{1}\;\text{Range}$	$\alpha_{2}\;\text{Range}$	Acceptable (Label: 1)	Unacceptable (Label: 0)	Total
Simulation	1 × 10^−3^–1 × 10^3^	0–5 × 10^2^	4410	7185	11595
Public phantom	1 × 10^−5^–1.9 × 10^3^	0–1 × 10^2^	576	580	1156
JHU phantom	1 × l^−5^–3 × 10^4^	0–3 × 10^2^	2554	2520	5074
In vivo breast mass	1 × 10^−5^–7 × 10^2^	0–1 × 10^2^	66	66	132
Training (Simulation & JHU Phantom Images)	1 × 10^−5^–3 × 10^4^	0–5 × 10^2^	5416	7985	13401
Testing (Simulation, Phantoms, In Vivo Images)	1 × 10^−5^–3 × 10^4^	0–5 × 10^2^	2190	2366	4556

**TABLE II:** Accuracy, Precision, Recall, and F1-Scores Achieved With the Proposed CNN

	Accuracy (%)	Precision (%)	Recall (%)	F1 score (%)
Single-inclusion simulated phantom	96.22	90.85	100	95.21
Multi-inclusion simulated phantom	93.92	89.09	100	94.23
Public phantom	99.45	98.96	100	99.47
JHU phantom	98.26	100	96.62	98.28
In vivo breast mass	77.34	68.1	100	81.04

**TABLE III:** RMSE and MAE Obtained From Single-Inclusion and Multi-Inclusion Simulated Phantoms

	RMSE		MAE	
	Single-inclusion	Multi-inclusion	Single-inclusion	Multi-inclusion
SOUL	7.66 × 10^−4^	1 × 10^−3^	4.69 × 10^−4^	6.32 × 10^−4^
AutoSOUL	7.97 × 10^−4^	9.85 × 10^−4^	5.12 × 10^−4^	6.73 × 10^−4^

**TABLE IV:** Median SNR, CNR, and SR Values Achieved With Test Datasets

	SNR		CNR		SR	
	SOUL	AutoSOUL	SOUL	AutoSOUL	SOUL	AutoSOUL
Single-inclusion simulated phantom	55.51	47.18	16.39	14.31	0.63	0.63
Multi-inclusion simulated phantom	44.80	31.31	20.87	16.48	0.54	0.52
Public phantom	25.47	22.62	13.28	13.06	0.53	0.50
JHU phantom	18.26	18.26	13.66	13.66	0.41	0.41
In vivo breast mass	19.87	22.38	10.37	10.46	0.49	0.52

## Highlights

- We introduced AutoSOUL, a deep learning-based framework to automate the selection of regularization parameters in energy-based ultrasound speckle tracking, to address manual tuning limitations.
- AutoSOUL classified strain images and recommended optimal parameters, closely matching manually tuned images in simulated, phantom, and in vivo breast mass datasets.
- AutoSOUL has the potential to improve efficiency and consistency in ultrasound elastography, considering the 84.4× time reduction for otherwise required manual parameter tuning and selection.

## Footnotes

1 Registered trademark.

2 Trademarked.
